# Sedation With Dexmedetomidine During Lymphangiography and Thoracic Duct Embolization in an Elderly Man

**DOI:** 10.7759/cureus.24466

**Published:** 2022-04-25

**Authors:** Rakuhei Nakama, Yasunori Arai, Tatsushi Kobayashi

**Affiliations:** 1 Department of Diagnostic Radiology, National Cancer Center Hospital East, Chiba, JPN

**Keywords:** elderly, dexmedetomidine, thoracic duct embolization, lymphangiography, procedural sedation, interventional radiology

## Abstract

An 83-year-old man underwent cervical esophagectomy and developed a chylothorax as a postoperative complication. We decided to perform lymphangiography and thoracic duct embolization for chylous leakage, but it was expected to be challenging to maintain bed rest. Therefore, dexmedetomidine was administered for procedural sedation. The patient’s blood pressure and heart rate were mostly stable during the procedure, and the sedation level was maintained within the desired limits. Due to its specific sedative pattern and mild analgesic effect, Dexmedetomidine is suitable for procedural sedation in various painless interventional radiology procedures, such as lymphangiography and thoracic duct embolization. Therefore, it may be the best sedative for the elderly and should be widely and effectively used in interventional radiology.

## Introduction

Interventional radiology (IR) is a minimally invasive procedure that can be safely performed without general anesthesia. Therefore, it may be safely used in elderly patients with complications. However, many IR procedures require a supine (or prone) position for an extended time and may require sedative medications. In addition, some IR procedures cause pain and require analgesia. Therefore, interventional radiologists use various medications to provide patients with comfort during IR procedures [1]. Dexmedetomidine (DEX) is a selective α2-adrenergic receptor agonist sedative often used in critical care medicine [2]. It has the advantage of having a very mild sedative effect and minimal respiratory depression. Therefore, it is easy to use this medication in the elderly, though there are few reports in the IR field. We present a case in which DEX enabled effective and safe sedation during lymphangiography and thoracic duct embolization in an elderly patient.

## Case presentation

An 83-year-old man underwent cervical esophagectomy, reconstruction with a free jejunal autograft, and gastrostomy for esophageal carcinoma. His clinical history included cerebral infarction and renal pelvis carcinoma (after total nephroureterectomy). A milky appearance was observed from the parajejunal drain on postoperative day three, indicating a chylous leakage. Generally, dietary limitation and octreotide administration are selected as initial non-invasive treatments for chylous leakage, but they may not be sufficiently effective or may take some time before the effect appears. Considering the patient's spare ability, interventional radiologists and surgeons discussed this case and decided to perform early lymphangiography and thoracic duct embolization for the chylous leakage on postoperative day eight. However, it was expected to be difficult for the patient to maintain bed rest as he had a mild delirium. Therefore, we attempted to use DEX for procedural sedation.

Lymphangiography was performed by puncturing the inguinal lymph node (23 G needle under ultrasound guidance) and gradually injecting iodized oil (Lipiodol, Guerbet Group, Villepinte, France). After the thoracic duct was sufficiently visualized using Lipiodol, we punctured the thoracic duct with a 21G needle under CT guidance and attempted catheterization. However, we decided to perform only lymphangiography because it was difficult to select the thoracic duct.

**Figure 1:**
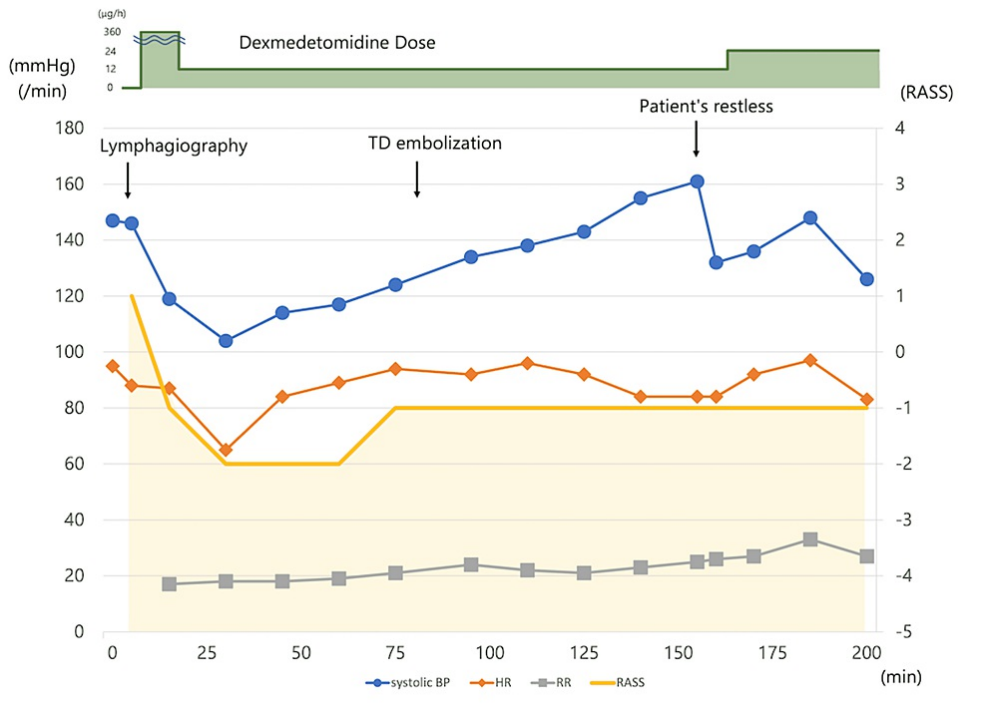
Clinical course in the angiography room. TD: thoracic duct; BP: Blood pressure; HR: heart rate; RR: respiratory rate; RASS: Richmond-Agitation-Sedation-Scale.

Figure 1 shows the degree of sedation and progress of the vital signs in the angiography room. The patient’s vital signs were stable when lymphangiography started, but the sedative level evaluated by the Richmond Agitation Sedation Scale (RASS) was +1 (anxious but movements not aggressive vigorous). DEX was started at a loading dose of 360 μg/h for approximately 10 min, and then the flow rate was changed to 12 μg/h. During lymphangiography and thoracic duct embolization, his blood pressure and heart rate were stable, and the sedation level was maintained within the desirable status, from RASS -1 (not fully alert, but has sustained awakening (eye-opening/eye contact) to voice) to -2 (briefly awakens with eye contact to voice). As the patient was restless during the thoracic duct embolization, the flow rate was increased to 24 μg/h, but no change in hemodynamics was observed. The total procedure time was 200 min, and all sedation procedures were performed by an interventional radiologist.

The chylothorax improved once but relapsed; therefore, thoracic duct embolization was performed anew under similar sedation on a postoperative day 13. No recurrence of chylothorax was observed after embolization.

## Discussion

We present a case of successful and optimal sedation with DEX during lymphangiography and thoracic duct embolization. We demonstrated that the elderly could be sedated conveniently without affecting their circulation or respiration.

DEX has already been widely utilized as an easy-to-use sedative. In particular, it is frequently administered in critical care and has been proven to reduce the duration of mechanical ventilation and length of ICU stay [3]. In radiology, it is used for sedation during pediatric diagnostic imaging [4]. However, there are few reports of its use in the field of IR.

Although the degrees of pain and length of time depend on the type of IR, there are no studies clarifying the suitability of DEX for specific types of IR. DEX has a mild analgesic effect mediated by the α2-adrenergic receptor [5-6]; however, additional analgesics may be required for IR associated with pain, such as uterine arterial embolization. DEX and analgesics are used in combination during embolization [7]. In such cases, considering that analgesics also have some sedative effects, the characteristics of DEX, which are “conscious sedation” and “co-operative sedation,” may not be clearly demonstrated [8].

DEX is also unsuitable for short procedures, such as image-guided biopsies, because it takes time to induce its effects. Accordingly, DEX may be suited for "painless" but "lengthy" procedures such as lymphangiography and thoracic embolization, as in this case. In fact, it has been reportedly effective during coil embolization for cerebral aneurysms [9].

Hence, DEX may be applicable to various types of IR. It alone can be sufficiently effective for sedation and analgesia during IR procedures.

## Conclusions

DEX may be suitable for painless but long time IR procedures, such as lymphangiography and thoracic duct embolization, and provide the elderly with safe sedation without respiratory depression. It may ease patients undergoing long-duration IR procedures without increasing adverse events. It is expected that the demand for IR, percutaneous procedures that do not require general anesthesia, will increase as the population ages in the future. DEX may be safe and suitable sedative for elderly patients and can be potentially useful in various IR procedures.
